# Preparation and morphology-dependent wettability of porous alumina membranes

**DOI:** 10.3762/bjnano.9.135

**Published:** 2018-05-15

**Authors:** Dmitry L Shimanovich, Alla I Vorobjova, Daria I Tishkevich, Alex V Trukhanov, Maxim V Zdorovets, Artem L Kozlovskiy

**Affiliations:** 1 Belarusian State University of Informatics and Radioelectronics, P. Brovki 6, Minsk 220013, Belarus; 2 Scientific and Practical Materials Research Center, Institute of Semiconductor and Solid State Physics, National Academy of Sciences of Belarus, P. Brovki 19, Minsk 220072, Belarus; 3 L. N. Gumilyov Eurasian National University, Abylaykhan, 2/1, Astana 010008, Kazakhstan; 4 The Institute of Nuclear Physics of Republic of Kazakhstan, Ibragimova 1, Almaty 050032, Kazakhstan; 5 Ural Federal University, Mira 19, Yekaterinburg 620002, Russia

**Keywords:** interfacial contact angle, membranes, porous anodic alumina, wettability

## Abstract

This article presents the preparation and study of the wetting properties of porous alumina membranes (PAMs) with a thickness of 25 to 75 μm and with a different pore sizes. The fabrication process features, scanning electron microscopy and atomic force microscopy characterization results are presented. The comparative analysis of PAM surfaces (outer and inner) and the effect of morphology of these surfaces on the wetting properties are discussed. Both alumina surfaces show significant morphology-dependent wettability. Measurements of the interfacial contact angle were made on the as-fabricated amorphous membrane and after pore widening with a range of pore diameters from 25 to 100 nm. The possible applications of PAMs for various membrane technologies is shown.

## Introduction

Porous anodic alumina (PAA) is increasingly attracting the attention of scientists due to its unique ordered honeycomb cell structure. Such a structure allows the formation of many new micro- and nanoelements via a template-assistant method [1–3]. In addition, PAA is an essential medium for carrying out unique scientific research. Many reviews and original papers have already been devoted to this material [4–8], and interest in PAM continues as a result of its new useful properties.

An actively developing field of materials science is the creation of new membrane materials. A new generation of membranes can be created using nanotechnology, which can increase the efficiency of their work by orders of magnitude [9]. The transition to a nanoscale devices requires the improvement of the most popular modern materials, such as polymer track membranes [10–11], and the development of new nanoporous composite materials based on PAA [12–13]. Both of these are commercially available. Porous silicon formed by electrochemical anodizing [14], zeolites [15], porous mica [16], nanoporous polymer glasses [17] and other materials [18] have also been studied as templates.

The disadvantages of polymer membranes include low chemical and thermal resistance and problems in regeneration. The maximum operating temperature of polycarbonate membranes is 450 K. PAA membranes in this respect are more preferable and have been intensively recently studied for use in biotechnological and medical development. Some applications include the separation of organic macromolecules and proteins (bio-filtration), their use in biosensor devices and capsule drug delivery systems, use for coating implants, and as a matrix for the formation of biocompatible tissues [19–22]. Membranes with a high selectivity and ordered structure can also be used to separate components of gas and liquid mixtures and to clean them from impurities (filtration). It is believed that in order to increase the selectivity and productivity, the membrane must have a regular structure of near-monodisperse cylindrical pores [23–24]. Membranes obtained by traditional methods have a three-dimensional pore structure that has a large pore size distribution, which does not allow high permeability values to be obtained [9,25–26]. Therefore, the optimization of parameters such as the diameter and length of the pores as well as the physical and chemical properties of the surface and walls of the PAM pores is a very urgent task.

No less important is the process of wetting the membrane with the depositing material (or its solution), depending on the variation of the template synthesis [27–28]. Recently membranes with special surface wettability have been investigated because of their potential application in microfluidics, self-cleaning and droplet-based technologies [19,29]. As shown in [30], by changing only the surface morphology of unmodified, bare PAMs, the wetting behavior could be altered from the Wenzel to the Cassie state.

The main aim of this study is preparation and investigation of the wetting properties of porous membranes with an ordered structure based on anodic alumina (PAMs) with different pore lengths and diameters. For this purpose we carry out a comparative analysis of the topological and technological characteristics on the resulting PAMs. The investigation of the wetting properties of PAM surfaces (outer and inner) was performed by measuring the interfacial contact angle (ICA). In our work, we show that with the control of morphology-dependent wettability of PAM it is possible to develop a template that is suitable for various membrane technologies.

## Experimental

In this study, various membranes were fabricated based on porous anodic alumina, prepared via a two-step anodization of Al foil using three distinct sets of conditions: type I – in an aqueous solution of oxalic acid (0.3 M H_2_C_2_O_4_) at 15 °C, 50 V; type II – in an aqueous solution of oxalic acid (0.3 M H_2_C_2_O_4_) at 15 °C, 40 V (as previously described in detail [31]); type III – in an aqueous solution of sulfuric acid (1.5 M H_2_SO_4_) at 15 °C, 20 V.

Before anodization, a technological frame was formed along the perimeter of the substrate. It is necessary to strengthen the mechanical stability of a free-standing membrane. The frame and its formation procedure are described in more detail in [32]. One further very important point related to the preparation of samples: as the membranes are almost transparent, it is advisable to designate the required surface of the PAM in advance.

**Figure 1:**
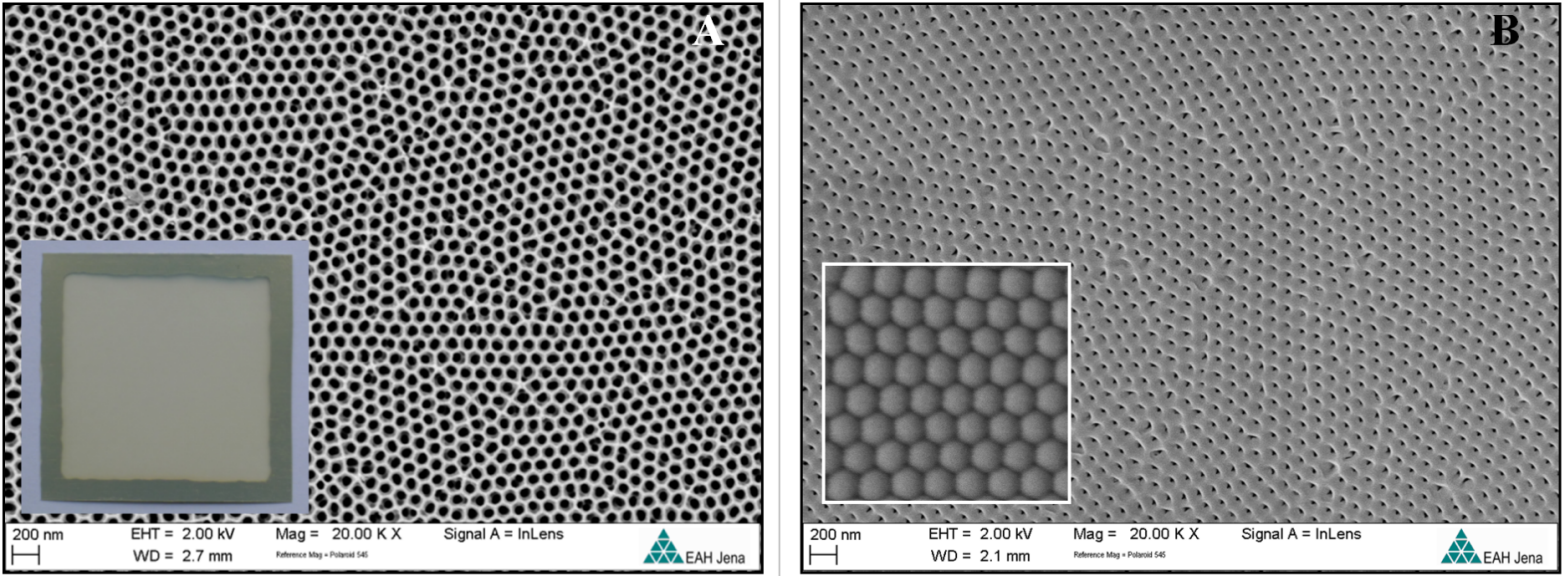
SEM images (after barrier layer etching) of a porous alumina membrane (PAM) fabricated using etch type I (with area 70 × 70 mm). The barrier layer was first opened on the back side using argon ions followed by the usual bilateral acid pickling. (A) top view (outer surface) and (B) top view (inner surface). The insets show optical images of the PAM and the enlarged SEM top view of the back side before barrier layer etching.

Further, the detachment of the alumina from the substrate was performed by Al dissolution in a saturated solution of cupric chloride and hydrochloric acid (HCl/CuCl_2_). Chemical dissolution of the barrier layer at the bottom of the pore and the chemical pore widening procedure was performed in 4 wt % Н_3_РО_4_ (35 °C) for different times. As a result, the porous alumina membrane (PAM) with ordered structure (Figure 1 and Supporting Information File 1, Figure S1) of 25–75 µm thickness with different pore diameters was obtained.

Five samples were prepared in total. For samples 1, 2, and 3 the etching of the barrier layer was carried out by immersing the entire membrane in a 4% aqueous solution of phosphoric acid (H_3_PO_4_) at 35 ± 2 °C for 15, 20 and 35 minutes, respectively. For the as-produced samples of type I and type II the barrier layer was not etched. For samples 4 and 5, the back side was first treated in argon plasma for 40 min and then in 4% H_3_PO_4_ at 35 ± 2 °C for 15 and 10 minutes, respectively.

The morphology of the experimental samples (pore diameter, *d*, interpore distance, *D*, and thickness, *H*), was examined by scanning electron microscopy (SEM, Philips, XL 30 S FEG and Hitachi, S-4800) and atomic force microscopy (AFM, Nanotop, NT-206 (Belarus) and Solver P47H, NT-MDT Co., Russia). Computer processing of the experimental data was carried out using the software package Surface Explorer Document (SED). This method allows for the study of the microstructure on a set of multiscale AFM and SEM images, covering a wide range of structure element size variations. The SEM images were also analyzed with the graphics editor Image J and ОriginPro8 software packages.

With any method of PAM fabrication there are two surfaces: the front (or outer) and back (or inner) surfaces. Preliminary studies have shown that these surfaces possess different physicochemical properties due to different degrees of hydrophilicity (as well as wettability). This concerns both industrially manufactured commercial membranes (e.g., PAM, nanochannel alumina NCA templates, Anodisc TM 25, Whatman Plc.) and domestically fabricated (in-house manufactured) PAMs.

The degree of PAM hydrophilicity was determined by measuring the interfacial contact angle (ICA), θ, by the recumbent drop method [33]. To do this, a drop of distilled water (≈15 μL) was applied to the surface of the samples from a microdoser. The ICA was determined by the goniometric method, in terms of the basic dimensions of the drop and the condition that θ < 90°, according to the equation

[1]
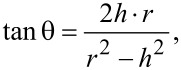


where, θ is the ICA, *r* is the radius of the contact area of the drop with the surface, and *h* is the height of the drop.

## Results and Discussion

### Topological features of porous alumina membranes

In the process of manufacturing of a PAM, the obtained membranes were analyzed after each main stage by means of SEM and AFM. Altogether several types of samples were prepared with different pore sizes and oxide thickness using various etching technologies of the barrier layer at the bottom of the pores (Table 1).

**Table 1:** Physical characteristics (average values) of the experimental samples (before etching, as-produced amorphous membrane).

Samples and forming conditions	Pore diameter, *d* (nm)	Cell diameter^a^, *D* (nm)	PAA thickness, *H* (µm)	Aspect ratio, *n* = *D*/*d*	Barrier layer thickness, *В* (nm)	Wall thickness, *W* (nm)

(type I) H_2_C_2_O_4_, 50 V	44	125 (115)	65	145	45.4	40.5
(type II) H_2_C_2_O_4_, 40 V	40	105 (110)	46	115	37.5	33.5
(type III) H_2_SO_4_, 20 V	25	45 (50)	75	300	15	14

^a^Calculated values are given with the mean values calculated based on SEM photos given in brackets.

**Figure 2:**
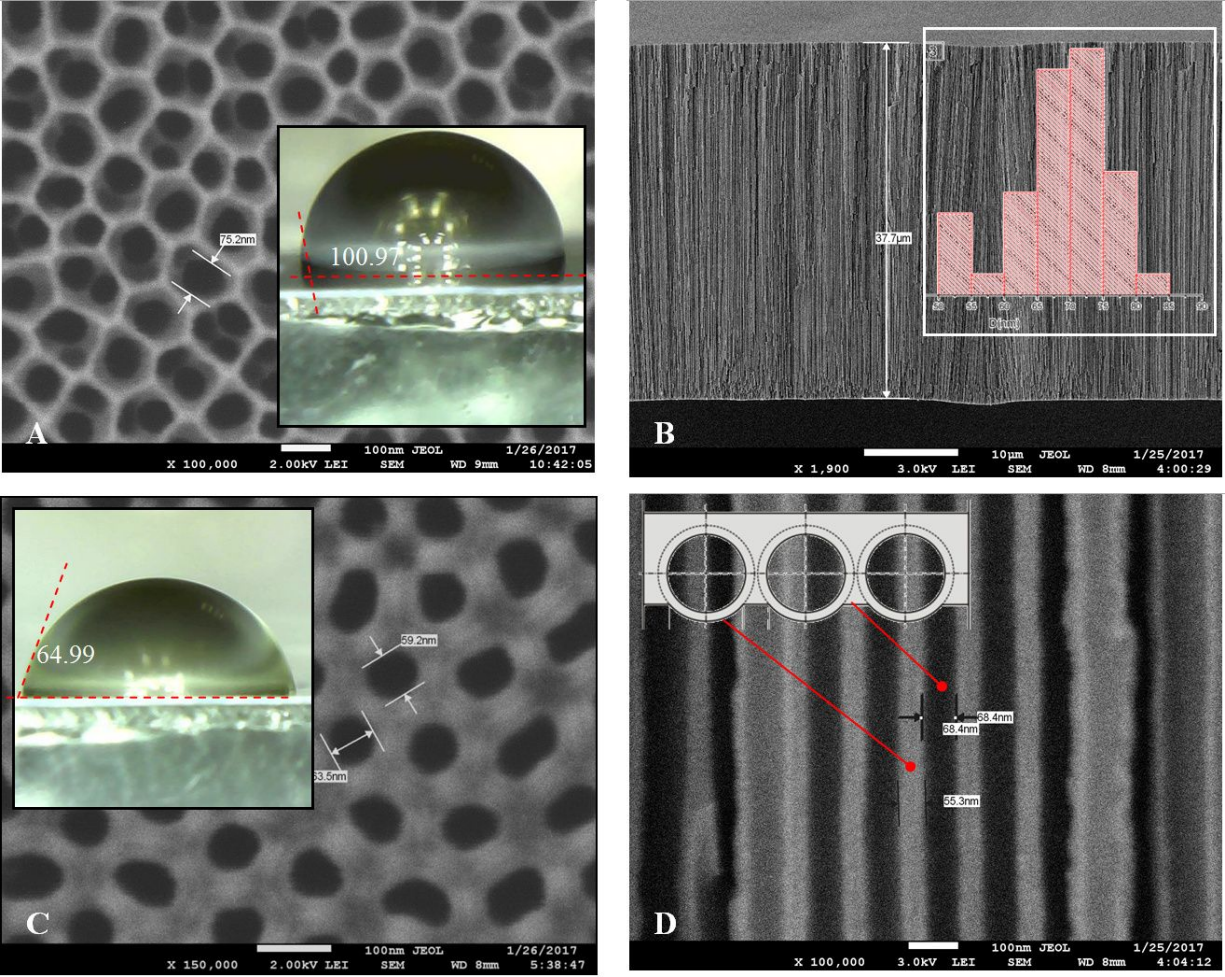
SEM surfaces (A, C) and cross-sectional (B, D) views of sample 2 (type I). In the insets – the contact angle images for the outer (A) and back (C) sides; (B) pore size distribution histogram; (D) the split surface formation scheme [34].

In Figure 2 the option of barrier layer etching on both sides (bilateral etching) by immersion of all free membrane in solution (sample No. 2) is shown. In the free membrane the aluminum layer beforehand is removed, as described previously.

In the inset of Figure 2D, the split surface formation scheme is shown [34]. This explains why the pore diameter in the SEM surface photo does not coincide with the pore size obtained in the SEM cross-sectional view of the same sample. In addition, mechanical damage to the fault surface can occur in the form of the fracture of the oxide cells while chipping. This disturbs the structural homogeneity and uniform orientation of the long channels in the membrane, which is fixed in the photographs.

Upon detailed study, it is evidenced that the various options for barrier layer etching are closely associated with the liquid distribution in the narrow channels (pores) of the PAM. Preliminary results show that a membrane thickness of less than 30 µm is enough to carry out an usual bilateral etching by immersion of the entire membrane into solution. At a membrane thickness of greater than 30 µm, it is desirable to first carry out the opening of the barrier layer on the back side using argon ions and then followed by the usual bilateral acid pickling.

**Figure 3:**
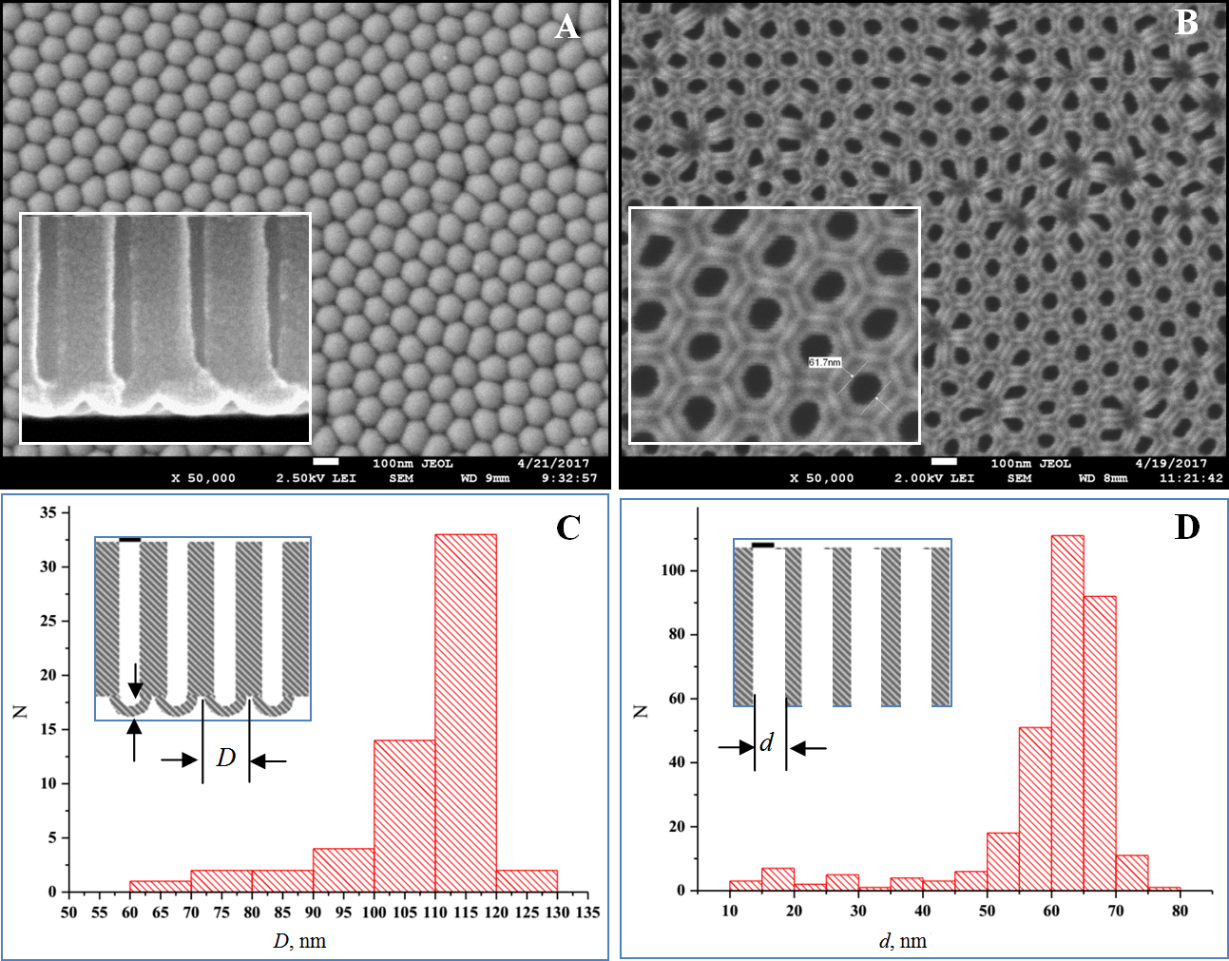
SEM images of the back surface of the sample before (A) and after (B) etching the barrier layer on the bottom of oxide pores. Histograms of distribution of the (C) oxide cell diameter (*D*) and (D) pore diameter (*d*), respectively.

To compare the topological features of PAMs produced with different etching technologies of the barrier layer, Figure 3 shows the membrane with a barrier layer on the back side.

Figure 3 shows SEM images of the back surface of a PAM before (A) and after (B) barrier layer opening on the bottom of the pores (the back side of the membrane). On the external boundary (in the inset of Figure 3A – scaled-up fragment of the chip), the edges of the porous oxide cells are clearly visible before the etching of the barrier layer. In the insets of Figure 3C,D schematically shown how the sample changes over the course of bilateral etching: the barrier layer is fully removed as a result of etching from two sides (shown by arrows), but the walls of the pore are only thinned as a result of etching only from within.

Theoretically, the distance between the pores (cell diameter) can be determined by the formula *D* = *k* × *U*_a_ ≈ 2.5 nm/V × (40 or 50) V = 100 and 125 nm, for type II (40 V) and type I (50 V), respectively. Here, *k* is a constant of proportionality for the diluted water electrolytes on the basis of acids and *U*_a_ is the voltage of anodization (i.e., forming voltage) for the Al foil [35]. The same values were found from the SEM images of the experimental samples (Table 1).

Table 1 lists other important physical parameters of the PAA structure: the thickness of the cell wall (*W*) and the thickness of the barrier layer (*B*). For highly ordered, densely packed, hexagonal PAA cells with a diameter of *D*, the cell wall thickness can be determined by the formula [36]: *W* = (*D* − *d*) / 2 = (125 − 44) / 2 = 40.5 nm. Besides, as we know, the wall thickness is related to the thickness of a barrier layer the following ratio [36]: *B* = 1.12 × *W* = 45.4 nm.

**Figure 4:**
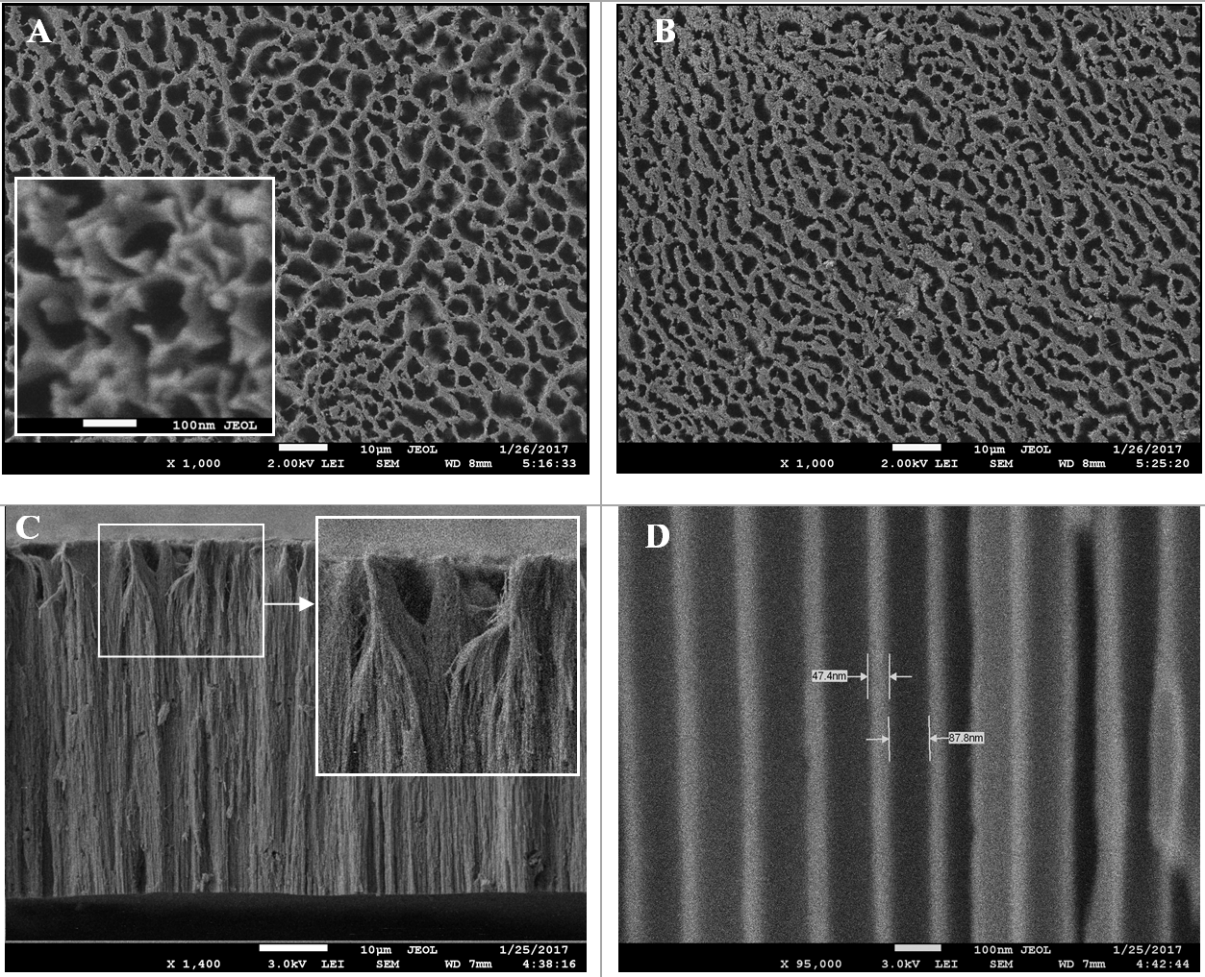
SEM images of the outer (A) and back (B) surfaces and cross-sections (C, D) of the sample (type I) after a complete etching of the barrier layer at the bottom of the oxide pores.

Figure 4 shows SEM images of surfaces and cross-sections of a PAM after complete etching of the barrier layer and partial etching of the pore walls ("etched" membrane) for the sample 3.

It can be seen from the Figure 4 that, in this case, the bottom and walls of the pore are dissolved at a different rate given this thickness of PAA (51.7 µm).

**Figure 5:**
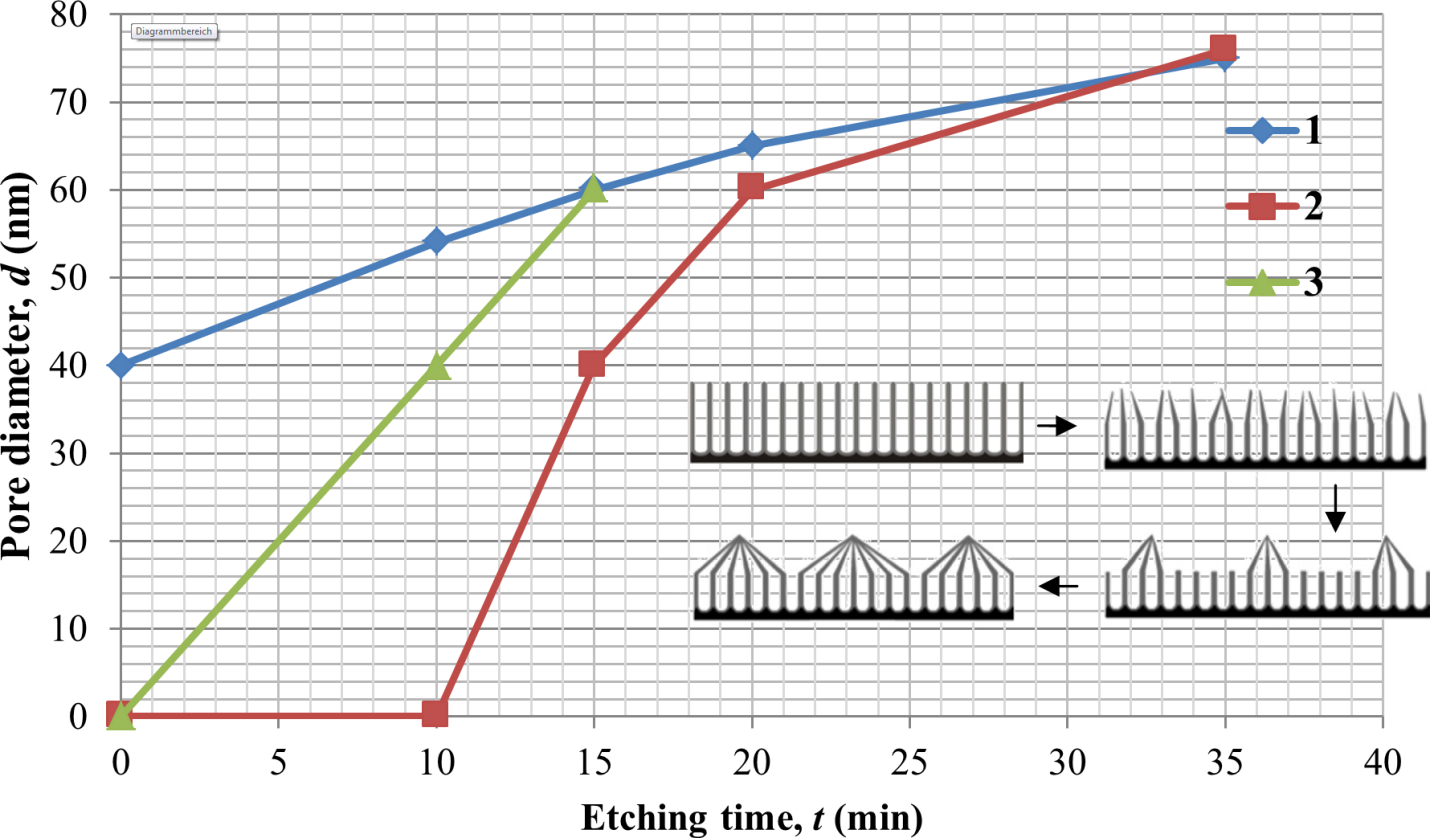
Pore diameter as a function of etching time of a barrier layer for the outer (1) and back (2, 3) surfaces of PAA, respectively: 3 – the back side was first treated in argon plasma for 40 min.

The cross-sectional photo in Figure 4C shows that the barrier layer near the internal border is dissolved a bit quicker than on walls. Therefore, the walls at the surface of PAA gather in bunches (see scaled-up fragment), as schematically shown in Figure 5 (scheme from [30]).

In Figure 5, the dependence of the pore diameter on etching time for the outer (data set 1) and back sides (data sets 2 and 3) of a PAM upon bilateral etching (using the usual method (1, 2)) are compared to the method including the pretreatment in argon media (3). The choice of an etching procedure in which both surfaces will be etched identically is practically very difficult as the reliefs of these surfaces are different from one another.

From Figure 4 it is evident that the solid barrier layer (without pores) is initially more quickly etched (the free access of solution to total surface). Then, in process of pore opening, the rates are counterbalanced. The physical and chemical properties of the barrier layer after pore opening do not change, but the structure of the channels and the conditions of solution flow in the narrow channels of a pore changes.

**Figure 6:**
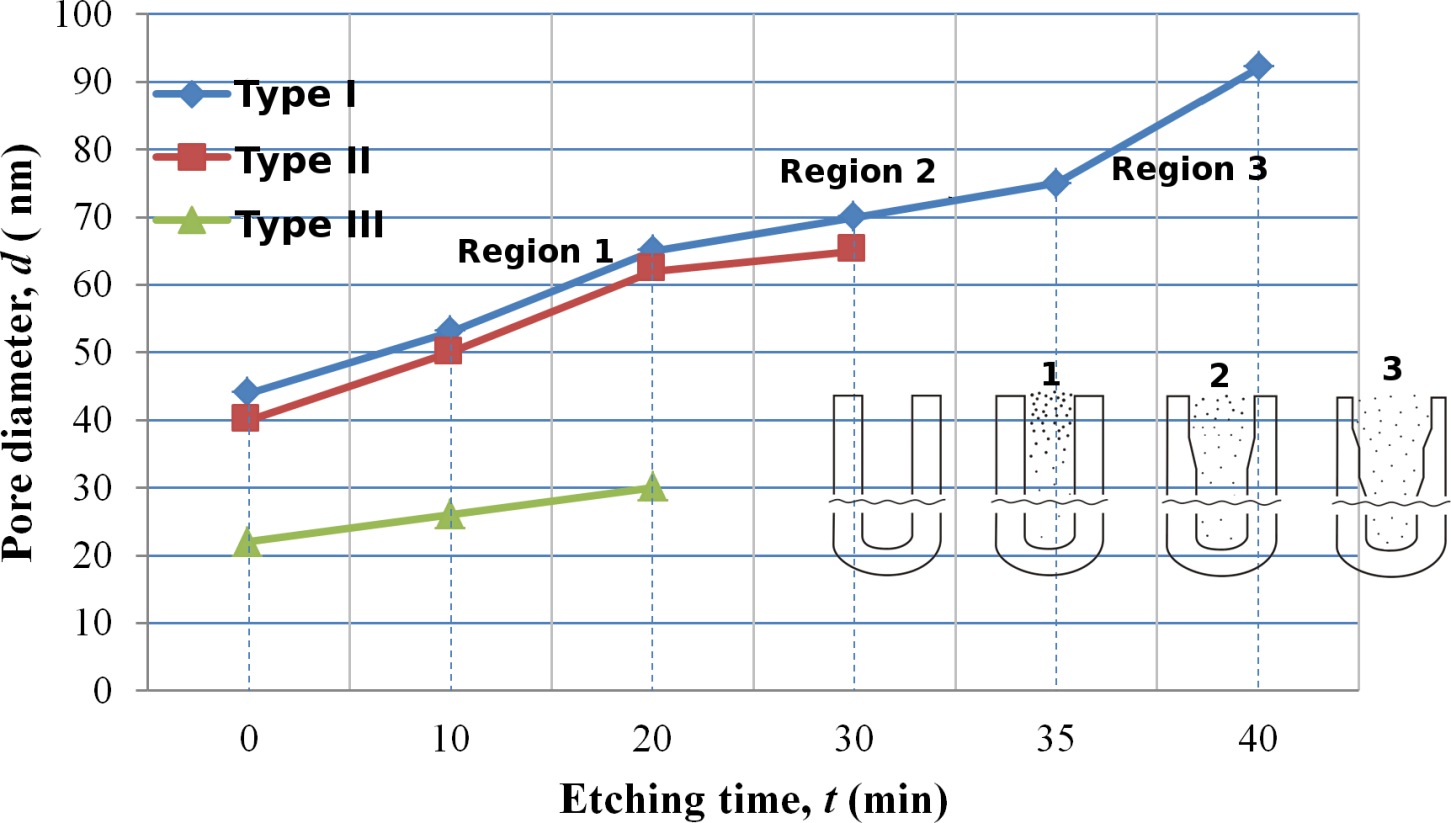
Pore diameter as a function of etching time of a barrier layer for PAA formed using the three synthesis condition schemes: Type I – in 0.3 M oxalic acid at a constant voltage of 50 V; type II – in 0.3 M oxalic acid at a constant voltage of 40 V; type III – in 1.5 M sulfuric acid at a constant voltage of 20 V. The temperature was held at 15 °C in all cases. The inset is a schematic describing the reaction occurring and correlating this to the three distinct trend regions (regions 1–3) for type I.

For the PAM prepared using type III conditions, a similar situation is observed (Supporting Information File 1, Figure S1A,B). The thinned cell walls (thickness ≈5 nm) gather in bunches on the surface, as shown in the SEM inset of Figure 4C, Supporting Information File 1 Figure S1B and schematically in Figure 6.

In Figure 6, the dependence of the pore diameter (outer surface) on etching duration of the barrier layer for PAMs formed under the various synthesis conditions explored in this work (type I–III) is shown. In the inset, a scheme describing non-isotropic etching of the pore walls [30] is shown. The authors of this work assume that the concentration of the electrolyte is initially higher on the surface of narrow channels (in the mouth of a nanopore, region 1). It then becomes almost uniform during their expansion (region 2). Therefore, the pore walls are etched non-uniformly (more quickly above), and as a result, gather in bunches on the PAM surface (region 3).

From Figure 6 it is also visible that the etching rate in the longest of the narrow channels (type III, PAM thickness of 75 μm, pore diameter of 25 nm, *n* = 300) is less than in the wider channels (type I and type II, PAM thickness of 50 and 65 μm, pore diameter of 40 and 44 nm, *n* = 145 and 115, respectively). The slope of curves on the initial line section increases with pore diameter for a PAM prepared in different electrolytes. Besides, after ≈35 min of etching, the pore diameter begins to increase more quickly, that is, the rate of etching increases. This can lead to erosion of the oxide and can lead to the loss of the shape-generating framework of the thin membrane (thickness <10 μm).

**Figure 7:**
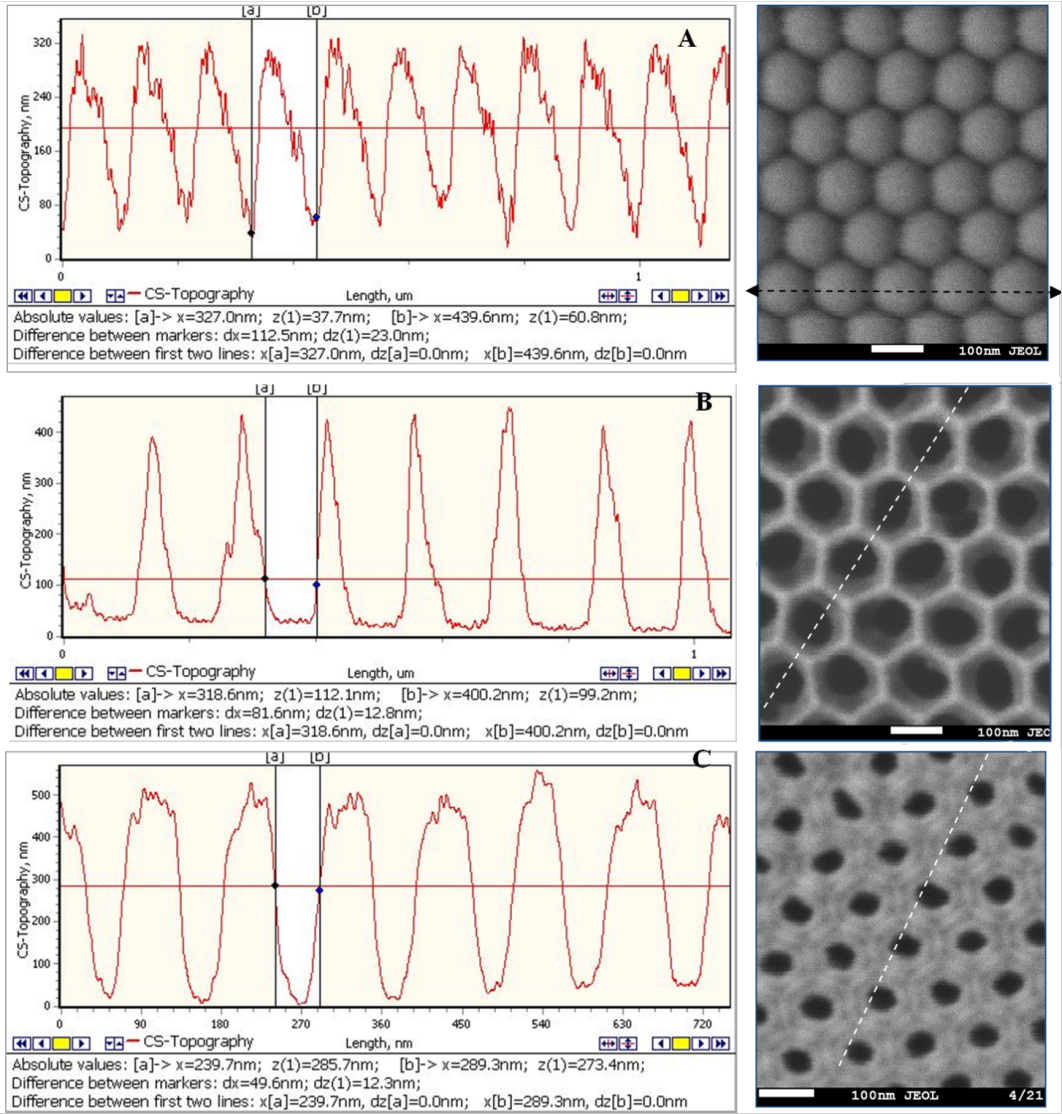
SEM images and AFM profiles of the back surface before (A) and after (C) etching of the barrier layer and the front surface (B) after etching of the barrier layer.

For assessment of homogeneity and pore size, Figure 7 shows the surface profiles of the experimental samples before and after barrier layer etching. Figure 7A,B shows the variation in pore diameter, and Figure 7C shows the distance between pores (cell diameter).

The pore diameter increased from 50 ± 5 nm to 80 ± 5 nm after 30 min of etching the barrier layer. The cell diameter did not change and the size is given in Figure 3A. The results of the AFM study are confirmed by the SEM data (the rate of etching corresponds to ≈1.0 nm/min)

Also, one of the most important parameters of a membrane, the porosity, depends on the pore diameter and structure of channels (and therefore, the penetration). The oxide porosity (with the through pores), α, was determined by expression the following equation [37]:

[2]
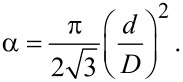


In general, the SEM images, AFM profiles and size histograms of the samples prepared using the optimum etching conditions show high uniformity of PAM topological parameters, both on the surface and in the middle of the sample (highly vertical orientation and high linearity of the nanochannels). Additionally, the results showed how both the outer and back surfaces of the PAM considerably changed, depending on the etching conditions.

### Wetting properties of porous alumina membranes

**Figure 8:**
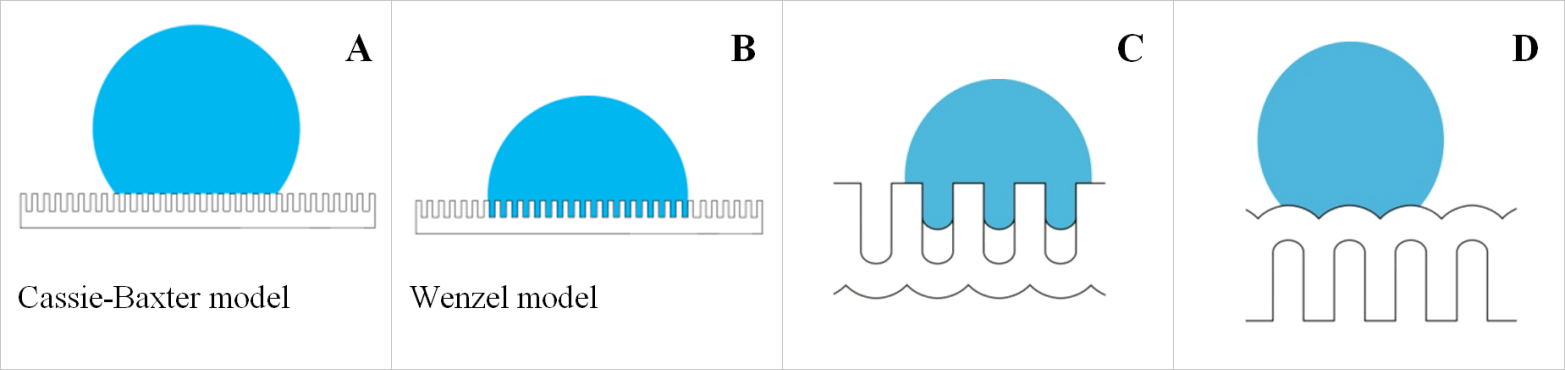
Schematic representation of the different models for pore wetting: A – Cassie–Baxter model [38], B – Wenzel model [38], C, D – schemes to explain wetting in our study.

The previously presented results show that the topological features of PAM surfaces can differ substantially depending on through-pores obtaining. Therefore, the contact (wetting) angle was determined on the outer and back sides of the membrane. Schematically views of water drop spreading are shown in Figure 8.

**Figure 9:**
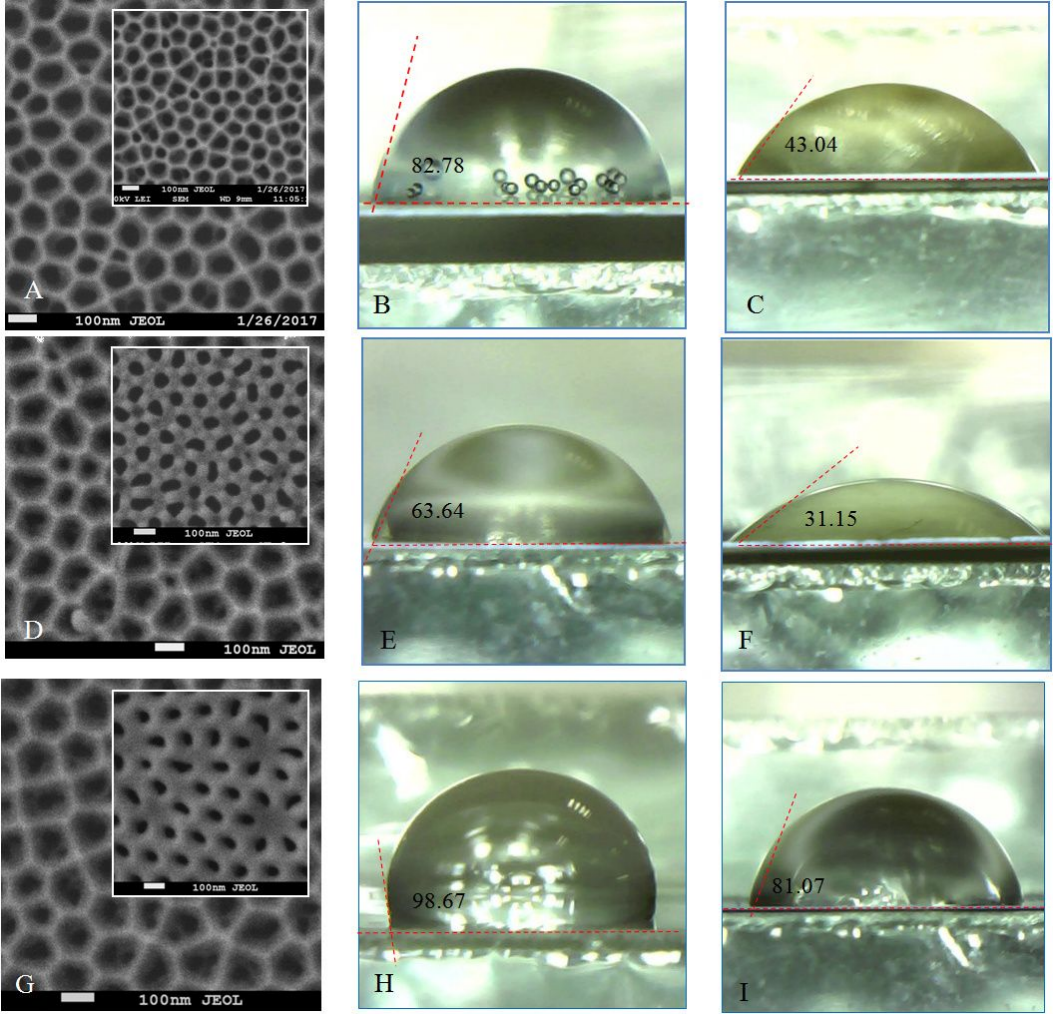
Contact angle as a function of surface topology (middle column images (B, E, H) are outer surfaces and right-hand column images (C, F, I) are back surfaces) for a PAM synthesized using type I synthesis conditions. (A–C) Sample 1; (D–F) sample 5 and (G–I) sample 4. (A, D, G) SEM images of outer and back (inset) surfaces of these samples.

In Figure 9, the contact angle images and SEM images of the outer and back surfaces of samples 1, 4 and 5 are shown.

Previously discussed models [38] cannot completely explain the experimental results presented in different reports. In addition to the contact angle, another highly influential factor is exerted by the physicochemical properties of a PAM surface, or as it is often called, the “surface chemistry”. In this study we did not change this factor so as to specify the intrinsic properties of the as-made amorphous membrane’s influence on the contact angle. In the literature, there have been no questions raised regarding this important point.

**Figure 10:**
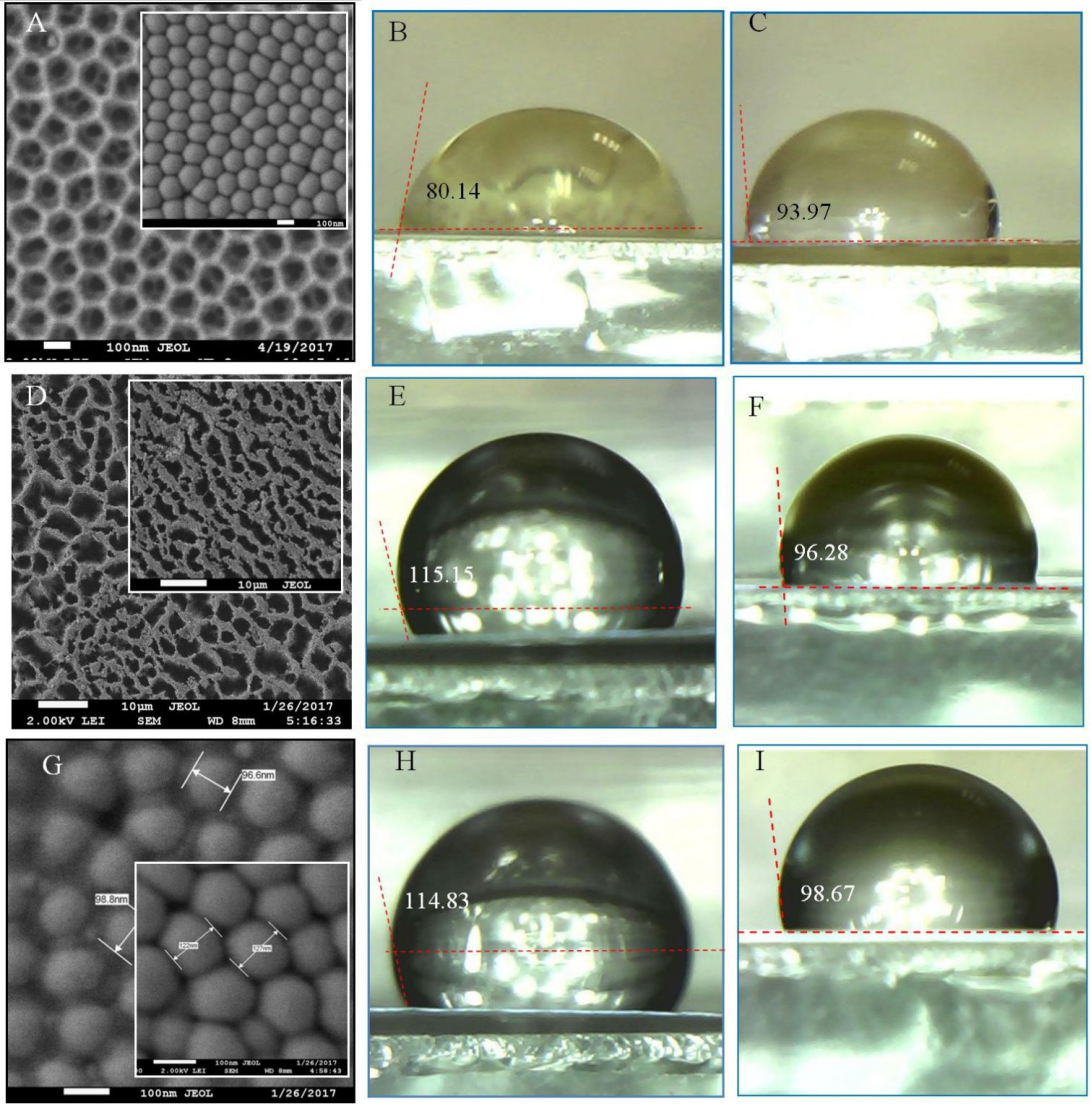
Contact angle as a function of surface topology (middle column images (B, E, H) are outer surfaces and right-hand column images (C, F, I) are back surfaces) for a PAM synthesized using type I synthesis conditions. (A–C) As-synthesized PAM; (D–F) sample 3. (A, D) SEM images of outer and back (inset) surfaces of these samples. (G) SEM images of the back side and the contact angle for the back side of a PAM (type I) before (inset) and after 7 min of chemical etching before pore opening.

In Figure 10, the contact angle images and SEM images of the outer and back surfaces of an as-made PAM (type I) (sample 3) and SEM images and contact angle images for the back side of PAM (type I) before pore opening are shown.

Several PAM parameters that are known to change over the course of the research timeframe are given in Table 2. The values of the contact angle for both the outer and back sides of the PAM are also given in Table 2.

**Table 2:** Contact angle values for as-made PAMs obtained in 0.3 M oxalic acid (type I and type II) and values for samples 1–5 after etching of the barrier layer (all of type I).

Sample	Pore diameter (outer side) (nm)	Pore diameter (back side) (nm)	Thickness (μm)	Porosity, α (%)	Contact angle (outer side), θ_1_ (degrees)	Contact angle (back side), θ_2_ (degrees)	Etch time^a^, *t* (min)

as-made (type I)	44	–	65	17	80.14	93.97	0
as-made (type III)	25	–	75	28	30.01	82.08	0
1	64	63	65	33	82.78	43.04	15
2	70	69	38	39	100.97	64.99	20
3	75	76	52	45	115.15	96.28	35
4	54	39	26	23	98.67	81.07	10^b^
5	50	60	45	20	63.64	31.15	15^b^

^a^Bilateral chemical etching of all substrates in 4% H_3_PO_4_ at 35 °C; ^b^Back side physical etching in argon for 40 min in addition to bilateral chemical etching of the whole substrate.

**Figure 11:**
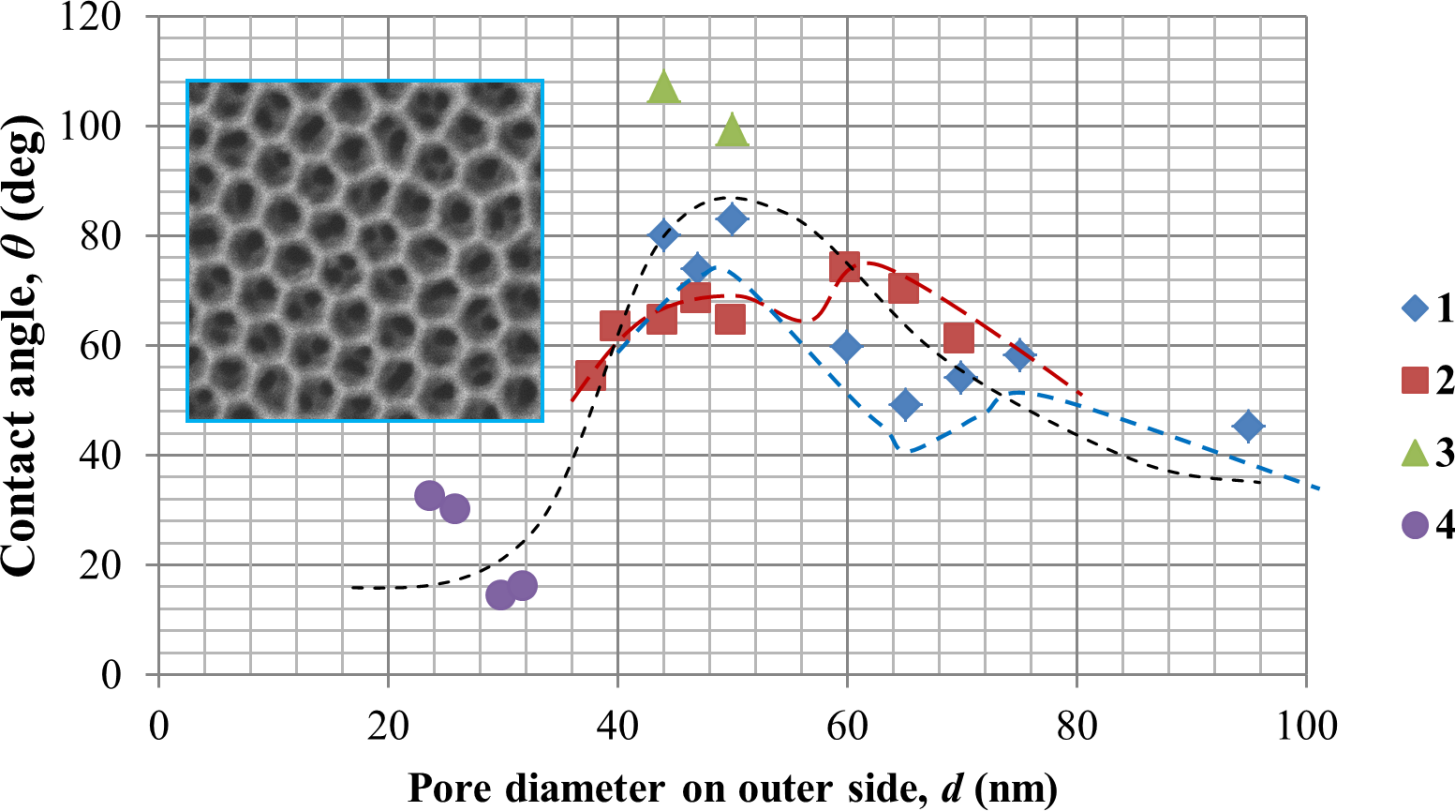
Contact angle measurements for the outer side of the PAM as a function of pore diameter for various membrane thicknesses: 1 (blue diamonds) – 28 µm; 2 (red squares) – 45 µm; 3 (green triangles) – 65 µm; 4 (purple circles) – 75 µm. The inset shows an SEM image of the outer surface. The black dotted line is a polynomial fit shown as a guide to the eye for all data sets.

**Figure 12:**
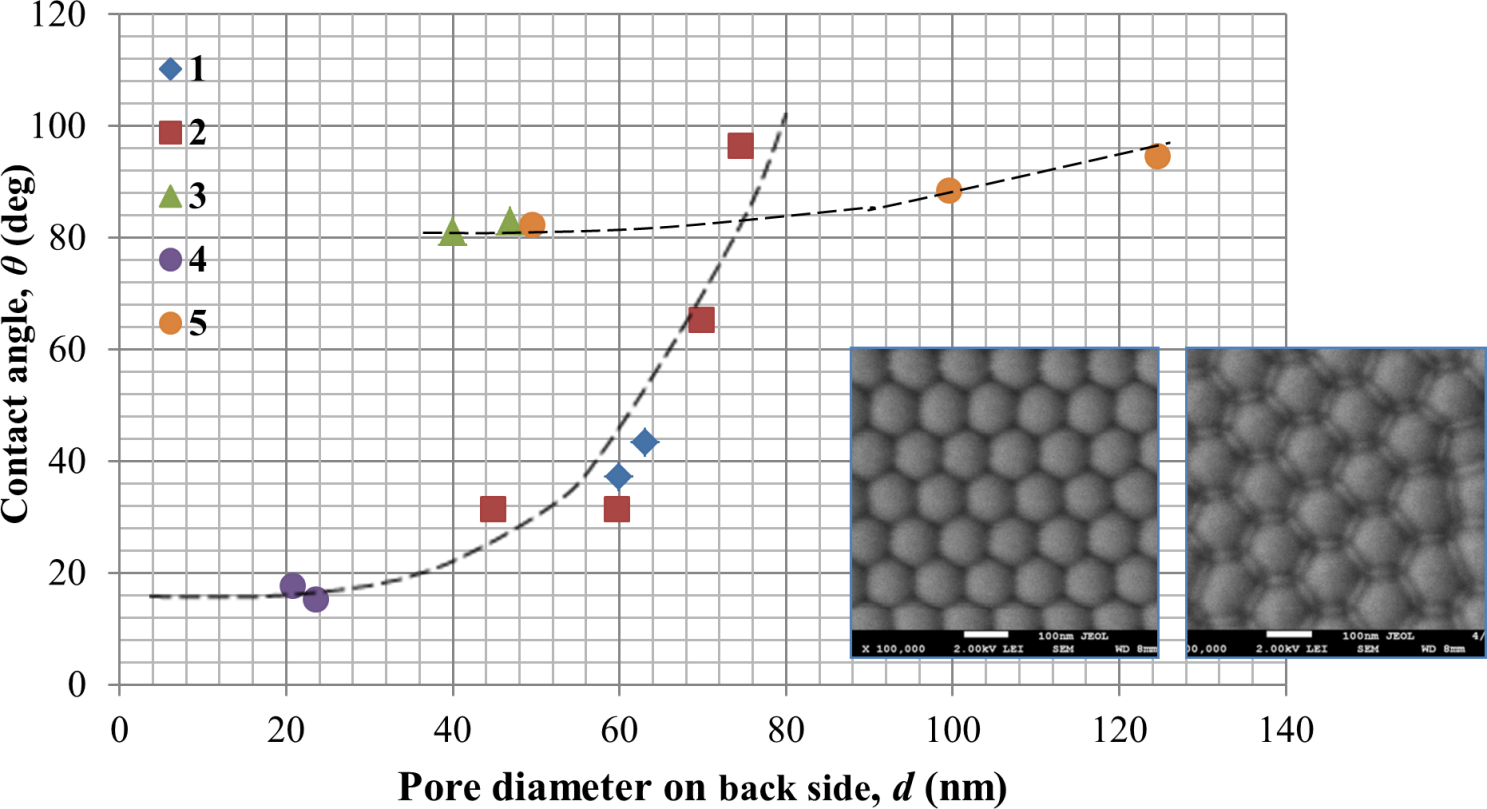
Contact angle measurements for the back side of the PAM as a function of pore diameter for various membrane thicknesses: 1 (blue diamonds) – 28 µm; 2 (red squares) – 45 µm; 3 (green triangles) – 65 µm; 4 (purple circles) – 75 µm; 5 – contact angle as a function of cell diameter for the as-made PAM (before pore opening). The inset shows SEM images of the back surface before (left) and after (right) chemical etching. The black dotted lines are polynomial fits shown as a guide to the eye for selected data sets.

In Figure 11 and Figure 12, the dependence of the wetting angle on pore diameter (outer and back surfaces) and cell diameter for PAMs of different thicknesses are shown.

From Figure 11 and Figure 12, it can be determined that the contact angle depends both on the pore diameter and PAM thickness. For the front side of the membrane, this dependence has several local maxima [39] that qualitatively and quantitatively can be correlated with results published prior works [40–41]. Initially, the contact angle increases with expansion of the pore diameter up to some conditional value (*d* ≈ 55 nm), after which it slowly decreases. The extrapolation of the contact angle values to *d* = 0 (i.e., for a smooth, porosity-free surface) leads to a contact angle of ≈18° that can also be correlated with the data of other publications [42].

The dependence on membrane thickness (or more specifically, the aspect ratio) is caused by specificity of solution flow in the narrow channels. More specifically, the solution flow conditions depend strongly on the structure of the porous medium. The specific pore size (or rather, the aspect ratio) depends on membrane thickness. A higher the aspect ratio results in restricted fluid flow. Therefore, the contact angle is also dependent upon this factor and thus how deeply the liquid (in this case, water) penetrates into the membrane pores. The dependence for the back side of the PAM (Figure 12) has a different character firstly due to the smaller pore diameter, and secondly, due to the completely different, non-planar relief of the surface (Figure 8D and inset of Figure 10G). The dependence on cell diameter is weak in this range. On such a surface, etching of the barrier layer occurs at the bottom of the pore as well as on the borders (joints) between the cells (Figure 10G), and probably at a different rate. It can be seen in the SEM images (insets) how changes in the interface occur over the course of etching before pore opening. Thus, it can be concluded that the PAM is not symmetric; it has a various relief structures for the front and back surfaces even given identical chemical treatment – i.e., etching in a 4% aqueous solution of H_3_PO_4_, рН 5.5 at 35 ± 2 °C.

From the given results, it can be concluded that the contact angle depends on the processing technique (back side of the membrane was processed in 4% H_3_PO_4_ and in argon plasma) and on the surface morphology. Therefore, differences in the measured contact angles and dependencies occur. A dependence on the membrane thickness is also observed, a fact that is not explained by any of the previous models of prior works.

## Conclusion

In this work, we have reported the preparation of porous alumina membranes (PAMs) by two-step anodization of aluminum foil in oxalic and sulfuric acid electrolytes. PAMs with a pore diameter in the range of 25 to 100 nm and 25 to 76 µm were fabricated. The anodization steps were performed in 0.3 M oxalic acid at 50 and 40 V and in 1.5 M sulfuric acid at 20 V. The surface chemistry of the PAM samples was specifically not modified in order to investigate only the effect of the native surface morphology on the ICA. It was shown that the contact angle depends not only on pore diameter, but also on PAM thickness. It was found that with the increase in etching time, the pore diameter and contact angle increased for both sides.

It was shown that it is possible to make membranes with hydrophilic properties, combining different methods of barrier layer etching. With the assistance of plasma treatment, the contact angle could be reduced by three times from 93.97° to 31.15° (see Table 2).

According to our results, it is possible to assume that the Cassie–Baxter model is more suitable for the description of large thickness oxides with small pores (received, for example, in sulfuric electrolyte). The Wenzel model was found to be more suitable for the description of small thickness oxides (less than 30 µm) with larger diameter pores (received, for example, in oxalate electrolyte).

The comparison of the wetting nature of the two surfaces of the PAM allows the contributions due to morphology and chemical properties to wetting of the nanostructure surface to be distinguished.

It was shown that the etching method influences the surface morphology of the PAM and, therefore, the contact angle. Thus, the front and back surfaces will result in non-homogenous reliefs, especially in the particular case of the etched membrane (see Figure 4), while the interior remains regularly porous.

This work demonstrates that porous alumina membranes with highly reproducible surface morphology and regular porosity can be fabricated using an inexpensive and handy technological process. Their remarkable and useful properties make PAMs promising substrates for various membrane technologies.

## Supporting Information

File 1 Additional experimental data.
SEM images of PAM (III) with an area of 70 × 70 mm and 75 µm thickness before and after barrier layer etching.
