# Supplementary material for: Preparation and morphology-dependent wettability of porous alumina membranes
Source: Beilstein J Nanotechnol. 2018 May 15;9:1423–36. doi: 10.3762/bjnano.9.135 (PMC6009415; doi:10.3762/bjnano.9.135)
Supplement: File 1 — Additional experimental data. SEM images of PAM (III) with an area of 70 × 70 mm and 75 µm thickness before and after barrier layer etching. [file Beilstein_J_Nanotechnol-09-1423-s001.pdf]

Supporting Information

for

Preparation and morphology-dependent wettability of porous alumina membranes

Dmitry L. Shimanovich[1], Alla I. Vorobjova[*1], Daria I. Tishkevich[2], Alex V. Trukhanov[2], Maxim V. Zdorovets[3,4,5], and Artem L. Kozlovskiy[3,4]

Address: [1]Belarusian State University of Informatics and Radioelectronics, P. Brovki 6, Minsk 220013, Belarus, [2]Scientific and Practical Materials Research Center, Institute of Semiconductor and Solid State Physics, National Academy of Sciences of Belarus, P. Brovki 19, Minsk 220072, Belarus, [3]L. N. Gumilyov Eurasian National University, Abylaykhan, 2/1, Astana 010008, Kazakhstan, [4]The Institute of Nuclear Physics of Republic of Kazakhstan, Ibragimova 1, Almaty 050032, Kazakhstan, and [5]Ural Federal University, Mira 19, Yekaterinburg 620002, Russia

[*] Corresponding author

Email: Alla I. Vorobjova - vorobjova@bsuir.by

Additional experimental data

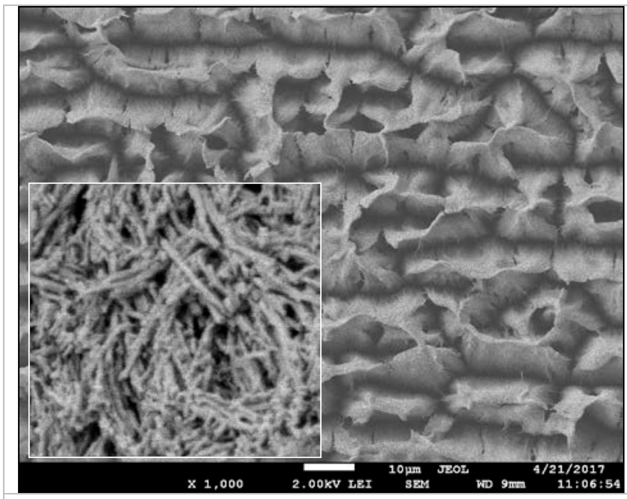

A: outer side after barrier layer etching

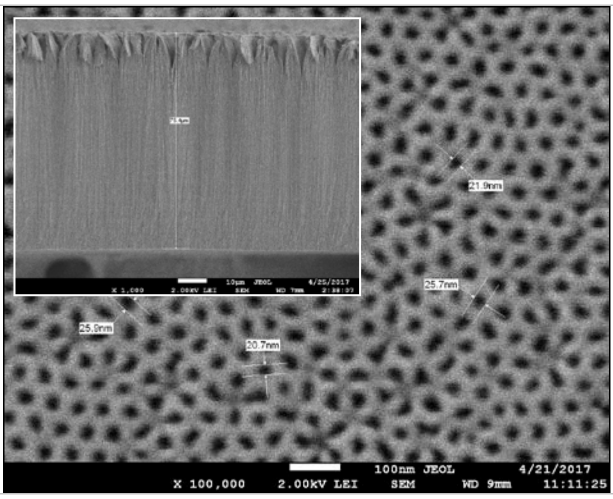

B: back side after barrier layer etching

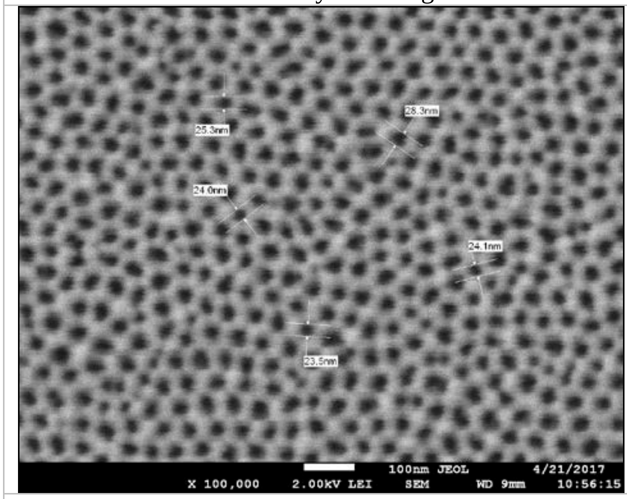

C: as made PAM (outer side)

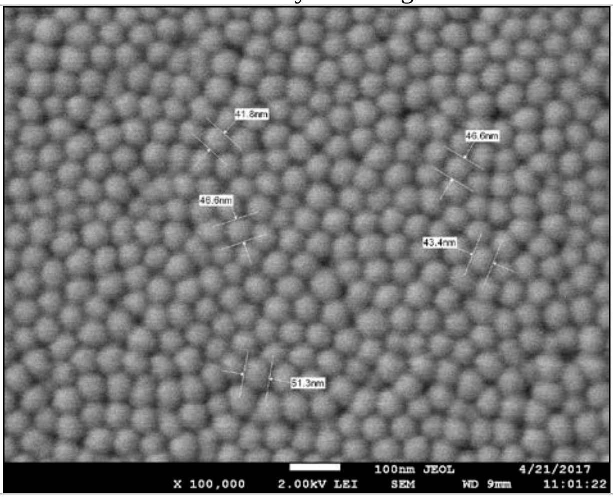

D: as made PAM (back side)

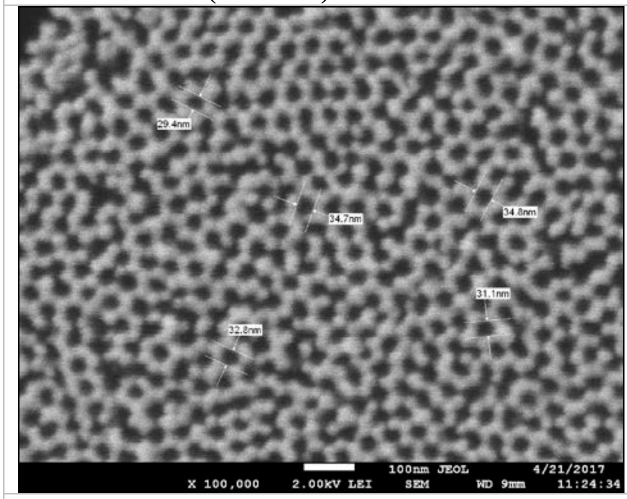

E: outer side after barrier layer etching

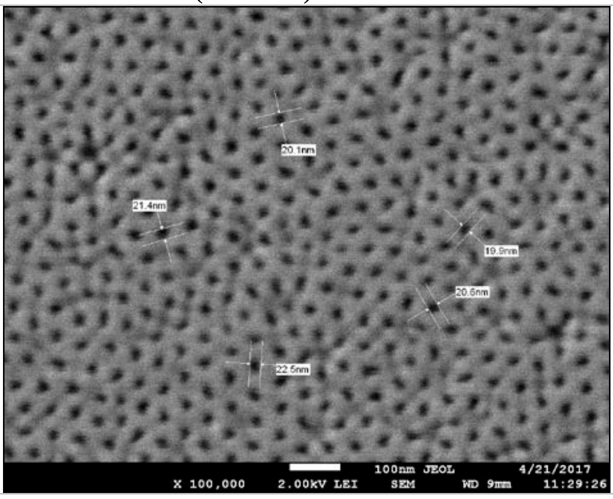

F: back side after barrier layer etching

Figure S1: SEM images of PAM (III) with size of 70 × 70 mm of 75 µm thickness before and after barrier layer etching.
